# Application of Suctioning Ureteral Access Sheath during Flexible Ureteroscopy for Renal Stones Decreases the Risk of Postoperative Systemic Inflammatory Response Syndrome

**DOI:** 10.1155/2022/9354714

**Published:** 2022-03-12

**Authors:** Xiaoyuan Qian, Chenqian Liu, Senyuan Hong, Jinzhou Xu, Can Qian, Jianning Zhu, Shaogang Wang, Jiaqiao Zhang

**Affiliations:** ^1^Department of Urology, Tongji Hospital, Tongji Medical College, Huazhong University of Science and Technology, Wuhan, China; ^2^Department of Traditional Chinese Medicine and Rheumatology, Southwest Hospital, Army Military Medical University, Chongqing, China; ^3^The Central Hospital of Wuhan, Tongji Medical College, Huazhong University of Science and Technology, Wuhan, Hubei, China

## Abstract

**Purpose:**

To clarify the efficiency and outcomes of suctioning ureteral access sheath (UAS) during flexible ureteroscopic lithotripsy (fURL) for the management of renal stones.

**Methods:**

Between January 2017 and January 2019, a total of 444 patients with renal stones undergoing fURL were divided into suctioning UAS and nonsuctioning UAS groups. The outcomes of patients in both groups were compared using a matched-pair analysis (1 : 1 scenario). Furthermore, a directed acyclic graph (DAG) was drawn to guide the multivariate logistic regression model and analyze the protective effect of suctioning UAS on the incidence of postoperative systemic inflammatory response syndrome (SIRS).

**Results:**

Before propensity score matching, significant differences were observed between the two groups in blood white cell counts, urine white cell counts, preoperative fever, preoperative indwelling stents, and laterality (*P* < 0.05). Eighty-one patients in the suctioning UAS group were successfully matched with 81 patients in the nonsuctioning group. The stone-free rate (SFR) on postoperative day 1 after fURL in the suctioning group was higher than that in the nonsuctioning group (86.4% vs. 71.6%; *P*=0.034), whereas it was comparable between the two groups 1 month after the surgery (88.9% vs. 82.7%; *P*=0.368). The incidence of postoperative fever or SIRS was lower in the suctioning group (fever: 3.70% vs. 14.8%; *P*=0.030; SIRS: 1.23% vs. 12.3%; *P*=0.012). However, the operative duration was similar in both groups (mean (SD)) (72.9 (28.1) min vs. 80.0 (29.5) min; *P*=0.121). The result of the multivariate logistic regression model guided by DAG revealed that the application of nonsuctioning UAS (odds ratio: 5.28 [1.38–35.07], *P*=0.034) during fURL was associated with postoperative SIRS.

**Conclusions:**

The application of suctioning UAS during fURL was associated with higher SFR on day 1 after surgery and a lower incidence of postoperative fever or SIRS.

## 1. Introduction

Kidney stones are one of the common diseases of the urinary system and have a high incidence and recurrence rate [1, 2]. If patients are not treated in time, the collection system is blocked, which can result in serious complications [2]. Gradually, the main therapeutic strategies [3, 4] have come to focus on minimally invasive therapy. Flexible ureteroscopic lithotripsy (fURL) has attracted more and more attention in the treatment of kidney stones given its advantage of being performed through a natural orifice of the human body and the low risk of direct damage to the kidney. However, fURL cannot actively discharge the crushed fragments. Patients take a long time to passively discharge the stones. Moreover, though some stones are crushed, they remain in the patient's body for a period of time, which predisposes them to urinary tract infections and stone recurrence.

The application of a ureteral access sheath (UAS) can accelerate the drainage of perfusion fluid, preventing high renal pelvic pressure (RPP) and postoperative infectious complications [5–7]; however, this pressure can only be decreased to a certain extent, especially for a ureteral sheath of a smaller diameter. Herein, the application of suctioning UAS is a good means of attaining a low RPP. Since the ureteroscopic water injection and drainage system can create a continuous circulation of water in the front of the ureteroscope, the perfusion fluid can be discharged more quickly. Therefore, the use of suctioning UAS can maintain low intraoperative RPP, make the surgical field clearer, and improve the efficiency of lithotripsy. However, whether the application of suctioning UAS is a crucial factor in reducing postoperative systemic inflammatory response syndrome (SIRS) remains undefined.

In this study, we compared the efficiency and outcomes between the suctioning UAS group and the nonsuctioning UAS group during fURL for the management of renal stones and verified the impact of the application of suctioning UAS on postoperative SIRS.

## 2. Materials and Methods

### 2.1. Study Population

Based on the tenets laid down by the Declaration of Helsinki, the Ethics Committee of Tongji Hospital, Tongji Medical College, Huazhong University of Science and Technology approved this study. Given the retrospective study design, the requirement for informed patient consent was waived.

A total of 444 patients who successfully underwent fURL for renal stones from January 2017 to January 2019 at our institution were retrospectively enrolled in this study. All patients underwent noncontrast computed tomography (CT) scans and were diagnosed with kidney stones. All patients who were older than 18 years and had undergone only fURL to treat unilateral stones were included. Patients with abnormal renal anatomy, infectious diseases, as well as incomplete perioperative data were excluded, as were patients with diseases that required intervention, such as stricture of the ureteropelvic junction or tumors requiring a biopsy. The criteria for fever and SIRS were as per the 2001 International Sepsis Definitions Conference definition. Postoperative fever was defined as an increase in temperature above 38.0°C within 48 hours after surgery. Systemic inflammatory response syndrome was defined when a minimum of two of the following four conditions occurred within 48 hours after fURL: (1) body temperature: <36°C or >38°C; (2) heart rate: > 90 beats/min; (3) respiratory rate: > 20 breaths/min or PaCO_2_: <32 mmHg; and (4) white blood cell count: > 12 × 10^9^/L or < 4 × 10^9^/L. Other causes leading to the abovementioned acute abnormalities were excluded [8].

Clinical data were collected from the electronic medical record management system. The patients' demographics were extracted. Preoperative data, including stone characteristics, blood and urine routine investigations, biochemical tests (albumin and globulin), and preoperative indwelling stent details were obtained. Intraoperative and postoperative information, including operation time, operation type, infectious complications, and SFR at postoperative 1 day and 1 month, were extracted.

Owing to the inherent imbalance in the baseline data and stone characteristics of patients between the suctioning UAS and nonsuctioning UAS groups, a 1 : 1 propensity score-matched analysis was used to adjust for differences in confounding factors. Each patient who underwent fURL with suctioning UAS was matched to another patient who underwent fURL without suctioning UAS. All patients were matched sequentially using the following covariates: sex; age; BMI; diabetes; hypertension; number and size of stones; laterality; white blood cell counts; urine white cell counts; urine culture; urinary nitrite; albumin and globulin levels; preoperative indwelling stent; and presence of preoperative fever.

### 2.2. fURL Procedure

All patients were placed in a low lithotomy position under general anesthesia. Rigid ureteroscopy was performed to dilate the ureter and a 0.035-inch flexible guidewire was inserted into the renal collecting system. Then, using the guidewire, UAS (12/14 F, Cook Medical) was inserted into the proximal ureter. After reaching the renal collecting system via the UAS, a 7.5-Fr flexible ureteroscope (Karl Storz SE and Co. KG, Tuttlingen, Germany) with a 200 *μ*m holmium YAG laser fiber whose energy was set at a range of 12–20 *W* and frequency of 14–20 Hz was used for laser lithotripsy. In the nonsuctioning group, the perfusion flow was adjusted to 50–100 mL/min. Larger stone fragments were picked out with a nitinol stone retrieval basket if necessary. In the suctioning UAS group, UAS was connected with the negative pressure pump whose pressure was maintained at 0.01 MPa. Meanwhile, the perfusion flow in the suctioning UAS group was set to 50–150 mL/min to fully discharge the residual fragments and perfusion fluid. Small stone particles were sucked out along the gap between the scope body and the suctioning UAS, while large stone fragments needed to be removed by slowly withdrawing the scope intermittently without the use of a stone basket, under direct vision. Finally, a 6-Fr ureteral stent was inserted at the end of the procedure to drain the urine.

### 2.3. Follow-Up

All patients underwent KUB or USG on postoperative day 1 to confirm the position of the indwelling double *J* tube and assess SFR. At one month postoperatively, patients underwent KUB or noncontrast CT imaging to evaluate SFR. The one-month postoperative data were obtained at the outpatient clinic. SFR was defined as either the complete absence of residual stones or the presence of asymptomatic residual fragments <4 mm in size.

### 2.4. Statistical Analysis

All statistical data were analyzed using the *R* version 3.6.3 (the *R* Foundation for Statistical Computing, Vienna, Austria). Continuous variables with a normal distribution were compared with a Student's *t*-test; otherwise, they were analyzed with a Mann–Whitney *U* test. Categorical variables were compared by the Chi-square test or Fisher's exact test. A directed acyclic graph (DAG) was created to illustrate confounder factors affecting the incidence of postoperative SIRS after fURL and intermediate variables excluded from the multivariable logistic regression model. A DAG-guided multivariable logistic regression model was created to analyze the impact of suctioning UAS on postoperative SIRS. A two-sided *P* < 0.05 was considered statistically significant.

## 3. Results

A total of 444 patients were enrolled in this study. Suctioning UAS was used in 81 patients, while the remaining 363 patients underwent fURL without suctioning UAS. The SFR was 77.7% on day one after fURL and 82.2% at one month postoperatively. SIRS occurred in 39 (8.78%) patients after fURL.

A statistically significant difference (*P* < 0.05) was observed between the two groups in blood and urine white cell counts, preoperative fever, preoperative indwelling stents, and laterality before propensity score matching. The other preoperative factors remained balanced (*P* > 0.05) between the two groups. Thus, 81 patients in the suctioning UAS group were successfully matched with 81 patients in the nonsuctioning group (Table 1). After matching was completed, the above-mentioned factors were similar (*P* > 0.05).

All patients underwent KUB one day postoperatively. One month postoperatively, 61.7% (50/81) of patients in the suctioning UAS group underwent noncontrast CT and 49.4% (40/81) of patients in the nonsuctioning UAS group underwent noncontrast CT (*P*=0.155), while the remaining patients underwent KUB to assess SFR. SFR on day 1 after fURL in the suctioning group was higher than that in the nonsuctioning group (86.4% vs. 71.6%; *P*=0.034), while no difference was observed one month postoperatively between the two groups (82.7% vs. 88.9%; *P*=0.368). The incidence of postoperative fever or SIRS was significantly lower in the suctioning group (fever: 3.70% vs. 14.80%; *P*=0.030; SIRS: 1.23% vs. 12.30%; *P*=0.036). However, no significant difference was observed in the operative time between the two groups (mean (SD)) (72.9 (28.1) min vs. 80.0 (29.5) min; *P*=0.121). The comparison of clinical outcomes is shown in Table 2.

**Figure 1:**
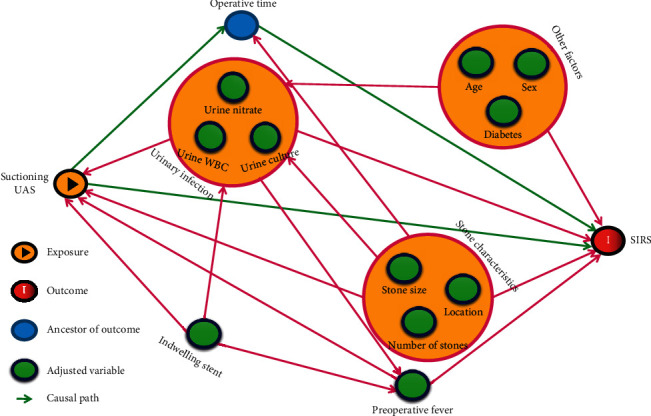
Directed acyclic graphs showing impact on postoperative SIRS of suctioning UAS.

Furthermore, to verify the effect of suctioning UAS on postoperative SIRS, a DAG was generated to explore the causal relationship between the application of suctioning and other variables while excluding intermediate factors confounding the study findings (Figure 1). Only operative time was recognized as an intermediate variable and was not included in the multivariate regression model. In the DAG-guided model, the nonsuctioning UAS group was significantly associated with postoperative SIRS (odds ratio, OR: 5.28 [1.38; 35.07], *P*=0.034). Besides, positive urine culture (OR: 3.60 [1.39–9.09], *P*=0.007), preoperative indwelling stent (OR: 5.72 [2.09–20.56], *P*=0.002), and stone size (OR: 1.07 [1.00–1.15], *P*=0.049) were also independent predictors of postoperative SIRS after fURL (Table 3).

## 4. Discussion

fURL does not cause direct damage to the patient's kidneys and allows a fast recovery; it is, therefore, a good alternative for the treatment of renal stones [4]. However, to visualize the intraoperative field clearly while breaking kidney stones and prevent injury to the renal collecting system, intraoperative saline perfusion is required, which results in elevated RPP. Postoperative infective complications often occur in cases of high RPP since it facilitates the entry of local bacterial organisms and endotoxins into the blood circulation [9, 10]. Herein, the use of suctioning UAS can lower RPP and enable clear intraoperative visualization [5]. Although suctioning UAS is increasing in popularity, studies on its impact on SFR and postoperative infective complications are limited. This study illustrates the impact of suctioning UAS on SFR and postoperative infective complications.

Though not statistically significant, we found that suctioning UAS was associated with a shorter operative time. The negative pressure provided by the connected vacuum suctioning tube can suck out the irrigating fluid, small broken particles, and pus moss in the renal pelvis through the sheath gap to keep the visual field clear and speed up the surgeon's operation [5]. Due to the negative pressure, the need for continuous perfusion for the crushing of the stone can be met. With a large flow rate, the crushed stones can be discharged quickly, which not only shortens the operative time but also improves the stone-breaking efficiency [5]. Moreover, with the rapid flow of irrigating fluid, crushed stones can be removed rapidly. Thus, we avoided the use of a stone basket in several patients during surgery and succeeded in shortening the operative time. It is commonly recognized that the duration of the procedure should be limited to approximately 1 hour. However, because operative time was an intermediate factor in the DAG-guided multivariate regression model, the effect of operative time on postoperative SIRS was not considered in the use of suctioning UAS.

Although SFR at one month postoperatively did not differ between the two groups, the suctioning UAS group had a higher SFR on postoperative day 1. Other studies including cases with consistent stone burden have also demonstrated a higher SFR on postoperative day 1 in the suctioning UAS group [6, 11]. One of the reasons may be that smaller gravel fragments are sucked out with perfusion after the stone is crushed by the holmium laser. Besides, a lot of dust is generated by the process of stone breaking, which may blur the operative field, making it difficult to distinguish small broken stone particles amidst the dust. Therefore, the application of suctioning UAS is of great significance to maintain a clear surgical field and to reduce the likelihood of residual stones. Additionally, traditional fURL lacks efficient stone clearance and mainly depends on stone basketing to remove the stone discontinuously, resulting in relatively low gravel efficiency. Moreover, short-term SFR is relatively restricted.

Infective complications were common after fURL [5, 6, 12], and our study found that the application of suctioning UAS could prevent postoperative fever or SIRS. High RPP has been shown to be related to postoperative fever, SIRS, or urosepsis [10, 13, 14]. In a retrospective study by Zhu et al. [6], the incidence of fever and urosepsis in the suctioning UAS group was lower and only accounted for 5.5%, whereas its incidence in the nonsuctioning UAS group was 13.9%. Another descriptive study by Deng et al. [5] obtained similar results and demonstrated that the application of negative pressure technology during fURL could decrease infective complications. The backflow level of RPP is usually 30 mmHg, and perfusion fluid in which bacteria and toxins are included can be absorbed into the circulation by pyelovenous-lymphatic backflow and pyelotubular backflow when intraoperative RPP is higher than the backflow pressure [5, 10]. Therefore, it is important to minimize intraoperative RPP to reduce regurgitation and the introduction of bacterial toxins into the bloodstream. Furthermore, the long narrow outflow channel of the ureteroscope can impede drainage, leading to high RPP. The application of negative pressure technique in endoscopic lithotripsy can ensure the right RPP, thereby reducing the incidence of postoperative infective complications reported in this study. To assess the impact of suctioning UAS on postoperative SIRS, we used DAG to exclude the effect of operative time which belonged to an intermediate variable on outcomes. Similarly, the application of suctioning UAS was related to postoperative SIRS and was protective against it.

Besides intraoperative factors, positive urine cultures, and preoperative indwelling stents were also found to be risk factors for postoperative SIRS. These risk factors for postoperative infective complications have often been reported in prior studies [15, 16]. Positive urine culture has been reported to be an important risk factor for postoperative infective complications after intraurological surgery [17, 18]. In this study, patients with a positive urine culture had a more than five-fold risk of postoperative SIRS, possibly attributed to elevated RPP promoting the entry of local infectious material including pathogens and toxins into the bloodstream and resulting in the occurrence of SIRS. Consistent with the finding of our previous [12], preoperative ureteral stenting could increase the risk of postoperative infective complications, which was verified in Shigemura et al.'s study [19]. For the indication of preoperative fever or urinary tract obstruction, most patients received a stent before surgery and often had infections caused by foreign bodies, which may be the main cause why patients in the stenting group had a higher rate of postoperative urosepsis.

The present study had certain limitations. First, our study had a retrospective study design, and the number of cases was relatively limited; a potential selection bias is also inevitable. Second, due to defects in the data collection process, a finite number of variables were collected, and several meaningful variables, such as stone culture, stent encrustation, and other postoperative complications such as septic shock, and renal pelvis/ureter perforation, were missing. Third, noncontrast-CT is more accurate than KUB or ultrasonography for the evaluation of SFR during follow-up; however, as per patients' preference, not all of them underwent noncontrast-CT during follow-up. Nonetheless, for the benefit of the patient, KUB or USG was performed one day postoperatively to evaluate SFR. Fourth, our system lacked a pressure monitoring and feedback system with the ability to monitor and adjust real-time RPP automatically. Therefore, high RPP may exist at times, which may affect the results to an extent. Finally, it needs to be emphasized that other postoperative complications, such as urosepsis, septic shock, and renal pelvis/ureter perforation, should have been included in our study but were not discussed in detail due to their low incidence at our institution. There is a need for larger prospective trials including more variables to validate our results.

The application of the negative pressure technique during fURL can reduce postoperative infective complications and improve the efficiency of lithotripsy to a certain extent, thus providing more possibilities for the treatment of relatively complex upper urinary stones. With the development of technology and improvement of existing equipment, fURL will largely replace percutaneous nephrolithotomy (PCNL) for the treatment of renal stones.

## 5. Conclusion

In conclusion, suctioning UAS enabled a higher SFR on postoperative day 1 and decreased postoperative infectious complications. Moreover, positive urine culture, preoperative indwelling stent, operative time, and nonapplication of suctioning UAS were risk factors for postoperative SIRS. Therefore, for some patients, the application of suctioning UAS can improve the occurrence of postoperative SIRS after fURL. To prevent the occurrence of postoperative SIRS, relevant preventive measures should be taken in the perioperative period.

## Figures and Tables

**Table 1:** Preoperative characteristics stratified by surgery type before and after propensity score matching.

Variables	Before propensity score matching			After propensity score matching		
	Nonsuctioning UAS group (363)	Suctioning UAS group (81)	*P* value	Nonsuctioning UAS group (81)	Suctioning UAS group (81)	*P* value
Sex, *n* (%)			0.806			0.617
Female	138 (38.0%)	29 (35.8%)		25 (30.9%)	29 (35.8%)	
Men	225 (62.0%)	52 (64.2%)		56 (69.1%)	52 (64.2%)	
Age (years)			0.740			0.883
Median (Q1, Q3)	49.0 (40.0, 58.0)	51 (42.0, 57.0)		50.0 (43.0, 57.0)	51.0 (42.0, 57.0)	
BMI (kg/m^2^)			0.512			0.735
Median (Q1, Q3)	23.7 (21.6, 25.7)	23.7 (22.1, 26.1)		24.2 (21.8, 26.3)	23.7 (22.1, 26.1)	
Diabetes mellitus, *n* (%)			0.243			1.000
Yes	35 (9.64%)	12 (14.8%)		12 (14.8%)	12 (14.8%)	
No	328 (90.4%)	69 (85.2%)		69 (85.2%)	69 (85.2%)	
Hypertension, *n* (%)			0.952			1.000
Yes	59 (16.3%)	14 (17.3%)		13 (16.0%)	14 (17.3%)	
No	304 (83.7%)	67 (82.7%)		68 (84.0%)	67 (82.7%)	
Albumin			0.220			0.827
Mean (SD)	40.7 (3.86)	41.2 (3.73)		41.1 (3.38)	41.2 (3.73)	
Globulin			0.934			0.785
Mean (SD)	-	-		28.1 (4.98)	27.9 (4.01)	
Median (Q1, Q3)	27.3 (24.9, 29.8)	27.8 (24.5, 31.2)		-	-	
AGR			0.410			0.796
Mean (SD)	1.48 (0.34)	1.51 (0.29)		1.50 (0.29)	1.51 (0.29)	
WBC			0.340			0.758
Median (Q1, Q3)	5.85 (4.89, 7.13)	5.86 (4.67, 6.84)		5.67 (4.77, 6.72)	5.86 (4.67, 6.84)	
Urine WBC, *n* (%)			<0.001			1.000
−	123 (33.9%)	62 (76.5%)		62 (76.5%)	62 (76.5%)	
+	240 (66.1%)	19 (23.5%)		19 (23.5%)	19 (23.5%)	
Urine culture, *n* (%)			0.197			1.000
−	320 (88.2%)	76 (93.8%)		76 (93.8%)	76 (93.8%)	
+	43 (11.8%)	5 (6.17%)		5 (6.17%)	5 (6.17%)	
Urine nitrate, *n* (%)			0.548			1.000
−	347 (95.6%)	79 (97.5%)		79 (97.5%)	79 (97.5%)	
+	16 (4.41%)	2 (2.47%)		2 (2.47%)	2 (2.47%)	
Preoperative fever, *n* (%)			<0.001			1.000
Yes	98 (27.0%)	3 (3.70%)		3 (3.70%)	3 (3.70%)	
No	265 (73.0%)	78 (96.3%)		78 (96.3%)	78 (96.3%)	
Hydronephrosis			0.391			0.278
Yes	332 (91.5%)	71 (87.7%)		5 (6.17%)	10 (12.3%)	
No	31 (8.54%)	10 (12.3%)		76 (93.8%)	71 (87.7%)	
Preoperative indwelling stent, *n* (%)			0.005			0.682
Absent	245 (67.5%)	68 (84.0%)		65 (80.2%)	68 (84.0%)	
Present	118 (32.5%)	13 (16.0%)		16 (19.8%)	13 (16.0%)	
Number of stones, *n* (%)			0.057			
1	202 (55.6%)	35 (43.2%)		43 (53.1%)	35 (43.2%)	
≥2	161 (44.4%)	46 (56.8%)		38 (46.9%)	46 (56.8%)	
Stone size (mm)			0.108			0.714
Median (Q1, Q3)	19.0 (16.0, 22.0)	19.0 (17.0, 23)		20.0 (17.0, 23.0)	19.0 (17.0, 23.0)	
Location			1.000			
Upper/middle pole	211 (58.1%)	47 (58.0%)		56 (69.1%)	47 (58.0%)	
Lower pole	152 (41.9%)	34 (42.0%)		25 (30.9%)	34 (42.0%)	
Laterality of stones, *n* (%)			0.001			1.000
Left	175 (48.2%)	22 (27.2%)		22 (27.2%)	22 (27.2%)	
Right	188 (51.8%)	59 (72.8%)		59 (72.8%)	59 (72.8%)	

BMI, body mass index; AGR, albuminglobulin ratio; WBC, white blood cell; fURS, flexible; UAS, ureteral access sheath.

**Table 2:** Comparisons of clinical outcomes between the two groups.

Index	Traditional fURL (81)	Suctioning fURL (81)	*P* value
Operative time (min)			0.121
Mean (SD)	80.0 (29.5)	72.9 (28.1)	
Postoperative SFR			
At 1 day	58 (71.6%)	70 (86.4%)	0.034
At 30 days	67 (82.7%)	72 (88.9%)	0.368
Postoperative infective complications			
SIRS	10 (12.3%)	1 (1.23%)	0.012
Fever	12 (14.8%)	3 (3.70%)	0.030

SFR, stone-free rate; SIRS, Systemic Inflammatory Response Syndrome; SD: standard deviation.

**Table 3:** DAG-guided multivariable logistic regression model analysis of causal effect of suctioning UAS on postoperative SIRS.

Variables	Multivariate	
	OR (95%CI)	*P* value
Sex		
Female	Reference	
Men	0.58 (0.28–1.21)	0.145
Age (years)	0.98 (0.95–1.01)	0.152
Diabetes mellitus		
No	Reference	
Yes	2.41 (0.81–6.55)	0.095
Urine WBC		
−	Reference	
+	1.22 (0.52–2.99)	0.651
Urine culture		
−	Reference	
+	3.6 (1.39–9.09)	**0.007**
Urine nitrate		
−	Reference	
+	1.07 (0.14–5.14)	0.941
Preoperative fever		
No	Reference	
Yes	1.18 (0.49–2.75)	0.703
Preoperative ureteral stenting		
Absent	Reference	
Present	5.72 (2.09–20.56)	**0.002**
Number of stones		
1	Reference	
≥2	0.96 (0.4–2.27)	0.918
Stone size (mm)	1.07 (1–1.15)	0.049
Location		
Upper/middle pole	Reference	
Lower pole	0.65 (0.29–1.39)	0.275
Surgery		
Suctioning UAS	Reference	
Nonsuctioning UAS	5.28 (1.38–35.07)	**0.034**

A two-sided *P*< 0.05 was considered statistically significant.

## Data Availability

The data supporting the findings of this study are available from the corresponding author upon reasonable request.

## Abbreviations

UAS: Ureteral access sheath
SFR: Stone-free rate
fURL: Flexible ureteroscopic lithotripsy
SIRS: Systemic inflammatory response syndrome
ESWL: Extracorporeal shock wave lithotripsy
PCNL: Percutaneous nephrolithotomy
RPP: Renal pelvic pressure
KUB: The abdominal kidney-ureter-bladder
CT: Contrast computed tomography
BMI: Body mass index
IQR: Interquartile range
SD: Mean ± standard deviation.

## Supplementary Materials

Supplementary Materials The Supplementary Table 1: the raw data from our manuscript “Application of suctioning ureteral access sheath during flexible ureteroscopy for renal stones decreases postoperative SIRS”.
