# Supplementary material for: Application of Suctioning Ureteral Access Sheath during Flexible Ureteroscopy for Renal Stones Decreases the Risk of Postoperative Systemic Inflammatory Response Syndrome
Source: Int J Clin Pract. 2022 Mar 12;2022:9354714. doi: 10.1155/2022/9354714 (PMC9159138; doi:10.1155/2022/9354714)
Supplement: Supplementary Materials — The Supplementary Table 1: the raw data from our manuscript “Application of suctioning ureteral access sheath during flexible ureteroscopy for renal stones decreases postoperative SIRS”. [file 9354714.f1.pdf]

The Supplementary table1: The raw data from our manuscript "Application of suctioning ureteral access sheath during decreases postoperative SIRS" .

No.	Age	Sex（1-man;0-female)	Diabetes(0-No;1-yes)	Hypertension(0-No;1-yes)	BMI	UWBC(0-Negative;1-positive)	Preoperative urine culture(0-Negative;1-positive)	Urine nitrate(0-Negative;1-positive)	Preoperative stenting((0-No;1-yes)	Number of stone (1-≥2;0 -1)	Size	Hydronephrosis(0-No;1-yes)	located in Low calyx(0-No;1-yes)	Preoperative fever(0-No;1-yes)	Laterality(0-right;1-left)	Operative time
1	18	1	0	0	21.60	1	0	0	1	1	30	1	1	0	1	75
2	18	1	0	0	24.21	1	0	0	1	0	23	1	0	1	1	75
3	18	0	0	0	18.07	1	0	0	1	0	17	1	1	1	1	50
4	19	1	0	0	22.13	0	0	0	1	0	16	1	0	0	1	50
5	19	1	0	0	20.96	0	0	0	1	0	16	1	0	0	1	45
6	20	0	0	0	18.73	0	0	0	1	1	17	0	1	0	1	90
7	20	1	0	0	21.22	0	0	0	0	1	31	1	1	0	1	50
8	20	1	0	0	18.21	0	0	0	1	0	18	1	0	0	1	40
9	20	1	0	0	21.85	1	0	1	0	1	30	1	0	1	1	50
10	21	1	0	0	21.22	0	0	0	1	0	15	0	1	0	0	36
11	21	1	0	0	19.72	1	0	0	0	0	21	1	0	0	1	105
12	21	1	0	0	22.46	1	0	0	1	1	21	1	1	0	1	55
13	22	1	0	0	27.72	0	0	0	0	1	19	1	1	0	0	116
14	22	0	0	0	22.51	1	0	0	0	1	17	1	1	1	1	45
15	22	1	0	0	22.60	1	1	0	1	0	15	1	1	0	1	35
16	23	1	0	0	22.04	1	0	0	1	0	12	1	0	0	0	40
17	24	0	0	0	24.66	0	0	0	1	0	9	1	0	0	1	90
18	24	1	0	0	26.57	0	0	0	1	1	19	1	0	0	0	64
19	25	1	0	0	19.05	1	0	0	0	1	17	1	0	1	1	70
20	25	0	0	0	20.31	0	0	0	1	0	20	1	0	0	1	48
21	26	1	0	0	28.33	1	0	0	0	0	13	1	0	0	1	50
22	26	1	0	0	23.57	1	0	0	1	1	23	1	1	0	0	115
23	26	0	0	0	27.61	1	1	0	0	0	15	1	0	0	0	80
24	27	1	0	0	21.80	1	0	0	1	1	23	1	0	0	0	80
25	27	0	0	0	18.29	1	0	0	1	0	14	1	0	1	0	55
26	28	0	0	0	25.39	0	0	0	1	1	19	0	1	0	1	50
27	28	1	0	0	25.35	0	0	0	0	0	22	1	0	0	0	90
28	28	1	0	0	24.91	0	0	0	1	0	17	0	0	0	0	60
29	28	0	0	0	25.24	1	0	0	1	1	27	1	0	0	1	120
30	29	1	0	0	23.53	1	0	0	0	0	19	1	1	0	0	70
31	29	1	0	0	23.88	1	0	0	1	0	9	1	1	1	0	35
32	29	1	0	0	17.44	1	0	0	1	0	12	0	1	0	1	45
33	29	1	0	0	23.39	1	0	0	0	0	15	1	0	0	0	43
34	30	1	0	0	24.22	0	0	0	1	1	26	1	1	0	0	55
35	30	0	0	1	17.91	1	0	0	0	0	14	1	0	1	1	100
36	30	1	0	0	27.55	1	0	0	1	0	15	0	1	1	1	45
37	30	0	0	0	36.05	1	1	0	1	0	16	1	0	1	0	39
38	30	1	0	0	22.39	1	0	0	1	0	19	1	0	0	0	72
39	31	1	0	0	20.38	1	0	0	1	0	12	0	0	0	0	45

40	31	1	0	0	21.88	1	0	0	1	0	10	1	1	1	1	60
41	31	1	0	0	28.34	0	0	0	1	0	10	1	0	0	1	30
42	31	1	0	0	21.80	0	0	0	1	1	20	0	0	0	1	60
43	32	0	0	0	22.58	0	0	0	1	1	18	1	0	0	1	110
44	32	1	0	0	23.92	1	0	0	1	1	27	1	1	0	0	110
45	32	1	0	0	21.80	0	0	0	1	0	20	1	0	0	0	75
46	32	1	0	0	20.70	1	0	0	0	1	26	1	0	0	0	120
47	32	1	0	0	18.26	1	0	0	0	0	15	1	1	1	0	190
48	32	0	0	0	16.89	1	0	0	1	0	20	1	0	0	1	60
49	32	0	0	0	22.15	1	1	0	1	0	10	1	1	1	0	100
50	33	1	0	0	23.44	0	0	0	0	1	27	1	0	0	1	90
51	33	1	0	0	23.05	0	0	0	1	0	17	1	0	0	1	51
52	33	0	0	0	22.22	1	0	0	1	1	23	1	0	0	1	40
53	33	1	0	0	25.71	1	0	0	0	0	16	1	0	0	1	67
54	33	1	0	0	22.53	1	0	1	0	0	17	1	0	0	0	50
55	34	1	0	0	26.42	1	0	0	0	0	15	1	0	0	0	47
56	34	1	0	0	22.72	1	1	0	1	0	20	1	0	1	0	55
57	34	0	0	0	25.35	1	0	0	1	1	16	1	1	0	0	60
58	34	1	0	0	24.38	1	0	0	1	0	25	1	0	0	0	73
59	34	1	0	0	27.78	1	0	0	1	0	20	1	0	0	0	130
60	35	0	0	0	23.24	0	0	0	0	1	19	1	1	0	1	95
61	35	1	0	0	25.64	0	0	0	1	0	20	0	0	0	0	120
62	35	1	0	0	26.78	0	0	0	1	1	16	1	1	0	0	120
63	35	1	0	0	22.65	1	0	0	1	1	22	1	0	0	1	116
64	35	1	0	0	22.49	0	0	0	1	0	20	1	0	0	1	92
65	35	1	0	0	28.23	1	1	0	0	0	15	0	0	0	0	45
66	35	0	0	0	21.88	1	0	0	0	0	16	1	0	0	0	81
67	35	0	0	1	22.77	0	0	0	1	1	17	1	1	0	0	60
68	35	0	0	0	20.06	1	0	0	1	0	19	1	0	1	1	73
69	36	1	0	0	25.83	0	0	0	1	1	24	1	1	0	1	120
70	36	0	0	0	25.10	0	0	0	1	0	10.5	1	0	0	0	90
71	36	1	0	0	24.82	0	0	0	1	1	19	1	0	0	1	55
72	36	1	0	0	22.06	0	0	0	1	1	17	1	1	0	1	50
73	36	1	0	1	30.45	0	0	0	1	0	18	1	0	0	1	43
74	36	1	0	0	23.03	1	0	0	0	1	22	0	0	0	1	80
75	36	1	0	0	19.03	1	1	0	1	1	28	1	1	1	0	20
76	36	0	0	0	21.23	1	0	0	1	1	21	1	0	0	0	74
77	36	0	0	0	20.58	1	0	0	1	1	19	1	0	0	1	40
78	36	1	0	0	22.84	1	0	0	1	1	23	1	0	0	0	35
79	36	1	0	1	23.88	1	0	0	0	0	18	1	1	0	0	57
80	37	0	0	0	24.09	0	0	0	0	0	8	1	1	1	0	40
81	37	0	0	0	19.98	1	0	0	1	1	26	0	1	0	1	118
82	37	1	0	0	28.41	1	0	0	1	0	12	1	0	1	1	55
83	37	0	0	0	19.29	1	0	0	0	0	15	0	1	0	1	59
84	37	1	0	0	25.24	1	0	0	1	1	29	1	1	1	1	130
85	37	1	0	1	21.97	0	0	0	1	0	19	1	0	0	0	107
86	37	1	0	0	21.26	0	0	0	1	0	20	1	0	0	1	49
87	38	0	0	0	22.48	0	0	0	0	1	27	1	1	0	0	50
88	38	1	0	0	26.64	1	0	0	1	0	13	1	1	1	1	40
89	38	1	0	0	21.10	1	1	0	0	0	10	0	1	0	1	95
90	38	1	0	0	24.22	1	0	0	0	1	19	1	1	0	0	60
91	38	1	0	1	22.02	1	0	0	1	0	21	1	0	0	1	70
92	38	1	0	1	26.84	0	0	0	0	0	19	1	0	0	1	50
93	38	0	0	0	19.05	0	0	0	1	1	15	1	0	0	0	60

94	38	0	0	0	19.83	0	0	0	1	0	12	1	0	0	0	50
95	38	0	0	0	24.22	0	0	0	1	0	16	1	0	0	0	50
96	39	0	0	0	27.84	1	0	0	0	1	23	1	0	1	1	50
97	39	0	0	0	18.90	0	0	0	1	1	30	1	0	0	1	69
98	39	1	0	0	13.03	0	0	0	1	1	19	1	0	0	1	95
99	39	1	0	0	20.20	0	0	0	1	0	17	1	1	0	1	79
100	39	0	0	0	26.03	0	0	0	1	1	19	1	0	0	1	90
101	39	1	0	0	24.21	0	0	0	1	0	16	1	0	0	1	90
102	39	0	0	0	24.22	0	0	0	1	0	10	1	1	0	0	100
103	39	1	0	0	33.80	0	0	0	1	1	20	1	0	0	0	55
104	40	1	1	0	26.29	0	0	0	1	0	15	0	0	0	0	60
105	40	0	0	0	21.19	0	0	0	1	0	19	0	0	0	1	53
106	40	0	0	0	19.14	0	0	0	1	0	16	1	0	0	0	55
107	40	0	0	0	20.23	1	0	0	1	1	27	1	0	0	0	130
108	40	1	0	0	24.25	1	0	0	1	0	22	0	0	0	1	40
109	40	1	0	0	27.76	0	0	0	1	0	16	1	0	0	1	90
110	40	1	0	0	23.18	1	0	0	1	0	27	1	0	0	1	55
111	41	1	1	0	25.95	1	0	0	1	0	21	1	1	0	1	40
112	41	1	0	0	29.30	0	0	0	1	0	24	1	0	0	1	55
113	41	0	0	0	26.44	1	0	0	1	1	19	1	0	0	0	70
114	41	1	0	0	29.14	0	0	0	1	0	8	1	0	1	1	30
115	41	1	0	0	30.44	0	0	0	0	0	9	1	0	0	0	30
116	41	0	0	0	25.39	0	0	0	1	0	8	1	0	0	0	45
117	41	0	0	0	28.73	1	0	0	0	0	15	1	0	1	1	35
118	42	1	0	0	25.86	1	0	0	1	1	18	1	0	0	1	30
119	42	0	0	0	25.10	0	0	0	1	0	15	1	0	0	1	40
120	42	0	0	0	22.86	0	0	0	1	0	15	1	1	1	1	105
121	42	1	0	0	24.21	1	0	0	0	0	23	1	1	0	0	83
122	42	0	0	0	23.53	1	0	0	1	0	17	1	0	1	1	120
123	42	0	0	0	23.01	1	0	0	1	1	29	1	1	0	0	120
124	42	1	1	1	24.09	1	0	0	0	1	20	1	0	0	0	75
125	43	1	0	0	27.34	1	0	0	0	0	12	1	1	1	1	60
126	43	1	0	0	21.45	1	0	0	1	1	20	1	1	1	1	110
127	43	1	0	0	21.63	1	0	0	0	1	30	1	0	1	1	70
128	43	1	0	0	23.46	1	0	0	1	1	20	0	0	0	1	72
129	43	1	0	0	25.91	0	0	0	1	1	10	1	1	0	1	96
130	43	0	0	0	27.55	1	1	0	1	1	20	1	0	1	0	60
131	43	0	0	0	25.07	1	0	0	1	1	27	0	1	0	0	120
132	43	1	0	0	21.48	1	0	0	1	1	22	1	1	0	0	70
133	43	1	0	0	22.89	1	0	0	1	0	17	1	1	1	1	63
134	43	1	0	0	24.34	1	0	0	1	1	26	1	1	0	1	100
135	43	0	0	0	18.99	1	0	0	1	1	19	1	1	0	0	60
136	43	0	0	0	23.44	1	1	0	1	1	18	1	0	1	1	60
137	43	0	0	0	25.35	0	1	0	0	0	8	1	0	1	1	30
138	44	1	0	0	19.53	1	0	0	0	1	32	1	0	1	0	60
139	44	0	0	1	21.88	1	0	0	0	0	9	1	0	0	0	70
140	44	0	0	0	21.63	1	0	0	1	0	25	1	1	0	0	60
141	44	1	0	0	23.88	0	0	0	1	1	17	0	1	0	1	70
142	44	1	0	0	24.84	1	0	1	1	1	24	0	1	0	1	160
143	44	0	0	0	24.50	1	1	1	1	1	24	1	1	0	1	110
144	44	1	0	0	26.30	1	0	0	1	1	30	1	0	1	1	90
145	44	1	0	0	26.64	0	0	0	0	0	15	1	0	0	0	56
146	44	0	0	0	19.23	1	1	0	1	1	17	1	1	1	1	44
147	44	1	0	0	21.97	1	1	1	0	0	9	1	1	1	1	75

148	44	1	0	0	21.30	0	0	0	1	0	16	1	0	0	1	55
149	45	0	0	0	23.83	1	0	0	0	1	28	1	0	1	1	140
150	45	0	0	0	20.96	0	0	0	1	0	14	1	1	0	0	60
151	45	1	0	0	25.83	0	0	0	1	1	21	1	0	0	1	63
152	45	0	0	0	20.63	1	0	0	1	1	22	1	1	0	0	70
153	45	0	0	0	19.38	1	1	0	0	1	23	1	1	1	1	70
154	45	0	0	0	20.73	1	0	0	1	0	15	1	0	1	1	75
155	45	1	0	0	23.44	1	0	0	1	0	19	1	1	0	1	60
156	45	0	0	0	32.46	0	0	0	1	1	26	1	0	0	0	90
157	45	1	0	0	26.04	1	0	0	1	0	8	1	0	0	1	55
158	46	1	0	0	20.76	0	0	0	1	0	18	1	0	0	0	54
159	46	1	0	0	32.87	1	0	0	1	1	18	1	1	0	1	60
160	46	1	0	0	28.01	0	0	0	1	1	27	1	1	0	1	61
161	46	0	0	0	20.81	1	0	0	1	1	35	1	0	1	0	100
162	46	1	0	0	24.34	0	0	0	0	1	21	1	0	0	0	116
163	47	1	0	0	18.59	0	0	0	0	1	20	1	0	0	1	62
164	47	0	0	0	20.76	1	0	0	1	0	13	1	1	0	1	80
165	47	1	0	0	24.57	0	0	0	1	1	22	1	1	0	0	157
166	47	0	0	0	19.10	0	1	0	1	0	16	1	0	0	0	40
167	47	1	0	0	16.26	1	0	0	1	1	20	1	0	0	1	69
168	47	1	0	0	23.05	0	0	0	1	1	21	1	1	0	0	120
169	47	1	0	0	32.18	1	0	0	0	1	21	1	0	0	1	40
170	47	0	0	0	29.07	1	0	0	0	0	15	1	0	0	1	55
171	47	1	0	0	22.92	0	0	0	1	0	14	1	0	0	1	52
172	47	1	0	0	23.18	1	0	0	1	1	29	1	1	0	1	150
173	47	1	0	0	24.86	1	0	0	1	1	19	0	1	0	0	60
174	47	1	0	0	26.87	0	0	0	1	1	19	1	1	0	0	75
175	47	0	0	0	22.65	1	0	0	0	0	10	1	0	0	1	40
176	47	1	0	1	27.72	1	0	0	1	0	15	1	0	0	1	45
177	47	0	0	0	23.18	0	0	1	0	0	8	1	0	0	1	35
178	48	1	0	0	21.09	1	0	0	1	0	11	1	0	0	0	40
179	48	1	0	0	24.91	0	0	0	1	1	20	1	1	0	1	90
180	48	0	1	0	30.30	1	0	0	1	0	20	1	0	0	1	60
181	48	1	0	0	21.51	1	0	0	0	0	22	1	0	0	0	60
182	48	1	0	0	25.83	1	0	0	0	0	12	1	0	0	0	55
183	48	0	0	0	21.63	1	0	0	1	1	24	1	1	0	1	120
184	48	1	0	0	24.03	1	1	0	0	1	11	0	0	0	0	50
185	48	1	0	0	23.04	1	0	0	1	1	33	0	0	0	0	150
186	48	1	0	0	22.48	1	0	0	1	0	16	1	1	1	1	70
187	48	1	1	0	29.75	1	0	0	1	1	29	0	1	0	1	170
188	48	1	0	0	23.84	0	0	0	0	0	15	1	0	0	1	45
189	48	1	0	0	21.22	1	0	0	1	1	26	0	1	0	1	45
190	48	1	0	0	23.88	1	0	0	0	1	15	0	1	0	0	50
191	48	1	0	0	20.96	1	0	0	1	0	16	0	0	0	1	70
192	48	1	0	0	28.37	1	1	0	1	0	18	1	1	1	0	110
193	48	1	0	0	25.69	0	1	0	1	0	12	0	1	0	1	30
194	48	1	0	0	15.67	1	0	0	0	1	25	1	1	0	1	100
195	48	1	1	1	23.73	1	0	0	1	0	18	1	0	1	0	135
196	48	1	0	0	23.51	0	0	0	1	0	16	1	1	0	1	99
197	48	1	0	0	16.81	1	0	0	1	1	30	1	1	1	1	55
198	48	1	0	0	23.59	1	1	0	0	1	17	1	1	0	1	77
199	48	0	0	0	21.63	1	0	0	0	1	19	1	1	0	1	55
200	48	1	0	0	18.83	0	0	0	1	0	20	1	0	0	1	90
201	49	0	0	0	23.51	1	0	0	0	1	18	1	1	1	0	52

202	49	0	0	0	22.41	0	0	0	0	0	10	1	0	1	0	55
203	49	1	0	0	23.92	1	1	0	1	1	20	1	1	1	1	75
204	49	1	0	0	20.96	0	0	0	1	0	17	1	0	0	1	38
205	49	1	1	0	25.65	1	1	1	1	0	18	1	1	1	0	70
206	49	0	0	0	20.03	0	0	0	1	1	17	1	1	0	1	55
207	49	1	0	0	31.16	0	0	0	1	1	25	1	1	0	1	140
208	49	0	0	0	13.67	0	0	0	1	0	18	1	0	0	1	17
209	49	1	0	0	26.64	0	0	0	1	0	26	1	0	0	1	55
210	49	1	0	0	22.23	1	0	0	1	0	17	1	0	0	0	103
211	49	1	1	0	26.26	0	0	0	0	1	24	1	1	0	1	99
212	49	1	0	1	23.32	1	0	0	1	0	18	1	0	0	1	67
213	49	0	0	0	21.48	0	0	0	0	0	20	1	0	1	1	36
214	49	1	0	0	18.37	1	0	0	1	1	21	1	0	0	1	180
215	49	0	0	0	22.83	1	0	0	0	1	18	1	1	0	0	20
216	49	1	0	1	26.67	1	1	0	1	0	23	1	0	1	0	165
217	49	1	0	0	24.84	0	0	0	0	1	29	1	0	0	0	120
218	49	0	0	0	27.10	1	0	0	0	0	21	1	1	0	1	90
219	50	1	0	0	26.59	0	0	0	0	1	24	1	1	0	1	126
220	50	1	0	0	30.48	0	0	0	1	1	28	1	0	0	1	100
221	50	1	1	0	24.16	1	0	0	1	1	28	1	0	0	1	63
222	50	0	0	0	17.09	1	0	0	1	1	18	1	0	0	0	90
223	50	1	0	0	27.68	0	0	0	1	0	21	1	0	0	1	90
224	50	0	0	0	28.44	0	0	0	1	0	18	1	1	0	1	60
225	50	1	0	0	18.37	0	0	0	1	1	25	1	0	0	1	60
226	50	1	0	0	23.74	0	0	0	1	0	21	1	0	0	1	64
227	50	1	0	0	23.05	1	0	0	0	1	23	1	1	0	0	110
228	50	0	0	1	26.67	1	0	0	1	1	20	1	1	0	1	75
229	50	0	0	0	21.56	1	0	0	0	0	24	1	1	0	0	120
230	50	0	0	0	24.61	1	0	0	1	1	20	1	1	0	1	50
231	50	1	0	0	24.51	1	0	0	0	0	21	1	0	0	0	105
232	50	0	0	0	27.01	0	0	0	1	0	16	1	0	0	1	53
233	51	1	0	0	26.81	0	0	0	1	1	20	0	0	0	1	65
234	51	1	0	1	25.10	0	0	0	1	1	26	1	0	0	0	40
235	51	0	1	0	26.49	1	0	0	1	1	25	1	1	0	0	135
236	51	1	0	0	25.69	1	0	0	1	0	18	1	0	0	0	65
237	51	0	0	0	17.01	1	0	0	1	1	19	1	1	0	1	45
238	51	1	0	1	22.78	0	0	0	1	0	17	1	0	0	1	95
239	51	0	1	1	26.06	0	0	0	1	0	20	1	0	0	1	67
240	51	0	0	0	21.51	1	1	0	0	0	18	1	1	1	0	110
241	51	0	0	1	23.37	0	0	0	1	1	23	1	1	0	0	60
242	51	1	0	0	23.14	0	1	0	1	0	17	1	0	0	0	60
243	51	1	0	1	26.12	1	0	0	1	1	19	1	1	0	0	70
244	51	0	1	0	24.84	1	1	0	1	1	24	1	1	1	0	60
245	51	1	0	1	29.76	0	0	0	1	1	24	1	0	0	1	117
246	51	1	0	0	23.67	0	0	0	1	0	17	1	0	0	0	70
247	51	0	0	1	25.86	1	0	0	1	1	17	1	0	1	1	75
248	51	1	0	0	22.27	1	0	0	1	1	23	1	1	0	1	88
249	51	0	0	0	24.44	1	0	0	1	0	13	1	0	1	1	35
250	51	1	0	0	24.34	1	0	0	1	0	14	1	1	1	0	60
251	51	1	0	0	22.40	1	0	0	1	1	22	1	1	0	0	65
252	51	1	0	1	25.97	1	0	0	1	0	20	0	1	0	0	45
253	52	0	0	0	25.31	1	0	0	1	1	32	1	0	1	0	100
254	52	1	0	0	22.84	1	0	0	1	0	21	1	0	0	1	55
255	52	0	0	0	22.86	1	0	0	0	1	25	1	0	0	0	75

256	52	0	0	0	24.61	1	0	0	0	1	26	1	0	0	0	81
257	52	1	0	0	24.17	0	0	1	1	0	19	1	0	1	0	65
258	52	0	0	1	24.22	0	0	1	0	1	27	1	1	0	1	106
259	52	1	0	0	25.95	0	0	0	1	1	26	1	0	0	1	128
260	52	1	0	0	21.87	1	0	0	1	1	19	1	0	0	0	75
261	52	0	0	0	17.48	0	0	0	1	1	26	1	1	0	1	110
262	52	1	0	0	20.34	0	0	0	1	0	18	1	0	0	1	60
263	52	1	0	0	21.95	0	0	0	1	0	15	1	0	0	1	50
264	52	1	1	0	25.35	0	0	0	1	0	20	1	0	0	1	60
265	52	1	0	0	23.05	0	0	0	1	0	17	1	0	0	0	66
266	52	1	0	1	27.68	0	0	0	1	0	17	1	0	0	1	105
267	52	1	0	0	25.61	0	0	0	1	0	18	1	0	0	1	55
268	52	1	0	0	28.44	1	0	0	1	0	19	1	0	0	1	55
269	52	1	0	0	20.70	1	0	0	1	0	12	1	0	1	1	40
270	52	0	0	0	24.06	0	0	0	1	0	20	1	0	0	0	67
271	52	0	0	0	19.98	1	1	0	1	1	29	1	1	1	1	155
272	52	1	0	0	26.56	1	0	0	0	0	10	1	1	1	1	65
273	53	1	1	0	19.96	1	0	0	0	1	17	1	0	0	0	85
274	53	1	0	1	24.80	0	0	0	1	0	22	1	0	0	1	65
275	53	0	0	0	25.18	1	0	0	1	0	16	1	0	1	0	85
276	53	0	0	0	26.37	1	1	0	0	1	29	1	0	1	0	60
277	53	0	0	1	37.46	1	1	0	0	0	8	1	0	1	1	95
278	53	1	0	0	26.37	0	0	0	0	1	28	1	0	0	0	55
279	53	1	0	0	23.50	1	0	1	0	0	16	1	1	0	1	75
280	53	1	1	0	24.22	1	1	0	1	1	21	1	1	0	1	70
281	53	0	0	0	21.63	1	0	0	1	0	12	0	0	0	1	25
282	53	1	0	0	25.10	0	0	0	1	0	18	1	0	0	1	85
283	53	0	0	0	22.48	0	0	0	1	1	18	1	0	0	1	68
284	53	0	1	1	22.43	1	1	0	1	0	21	1	0	0	0	60
285	54	1	1	1	27.72	1	0	0	0	0	16	1	1	0	1	75
286	54	1	0	1	24.34	1	0	0	0	1	22	1	1	1	0	65
287	54	1	1	0	26.03	0	0	0	0	1	14	1	1	0	0	187
288	54	0	0	0	19.53	1	1	0	0	1	24	1	1	0	1	90
289	54	1	0	0	23.66	0	0	0	1	1	22	0	1	0	1	70
290	54	1	0	0	22.04	0	0	0	1	1	27	1	1	0	1	55
291	54	0	1	0	22.86	0	1	0	1	0	19	1	0	0	1	105
292	54	1	0	0	30.82	1	0	0	0	0	19	1	1	0	0	61
293	54	0	0	0	23.31	1	0	0	0	0	10	1	0	0	1	75
294	54	1	0	1	26.06	0	0	0	0	1	31	1	0	0	0	165
295	54	1	0	1	26.57	1	0	0	0	1	24	1	0	0	1	60
296	55	1	0	0	29.22	0	0	0	1	1	18	1	0	0	1	60
297	55	0	0	0	20.57	1	0	0	1	1	19	1	1	0	0	70
298	55	1	1	0	24.54	0	0	0	1	1	21	1	0	0	1	96
299	55	0	0	0	25.97	1	1	0	1	1	19	1	1	0	0	66
300	55	0	0	0	23.83	1	0	0	1	0	19	1	0	0	0	70
301	55	1	0	0	17.72	1	0	0	1	1	20	1	1	0	0	60
302	55	0	0	0	26.45	1	0	0	1	0	20	1	0	1	1	110
303	55	0	0	0	27.48	1	0	0	1	0	19	1	0	1	1	45
304	55	0	1	0	20.45	1	1	0	0	0	26	1	0	1	1	101
305	55	1	0	1	21.48	1	0	0	1	1	22	1	1	0	1	75
306	55	0	0	0	18.37	1	1	1	1	1	28	1	1	0	0	120
307	55	0	0	0	17.99	1	0	1	1	0	11	1	1	1	0	80
308	55	1	0	0	25.33	1	0	0	1	0	11	0	1	0	1	30
309	55	1	0	0	22.77	1	0	0	0	0	16	1	1	0	0	49

310	55	1	0	1	30.04	0	0	0	1	1	29	1	1	0	0	80
311	56	1	0	0	22.72	1	0	0	0	0	17	1	0	0	0	60
312	56	1	0	1	26.73	0	0	0	1	1	19	1	0	0	0	50
313	56	1	0	0	25.28	0	0	0	1	1	18	0	0	0	1	45
314	56	0	0	1	27.64	0	0	0	1	1	29	1	1	0	1	50
315	56	1	0	0	21.68	1	0	0	1	0	8	0	1	0	1	45
316	56	1	1	1	24.77	0	0	0	0	0	15	1	0	0	1	55
317	56	0	0	0	26.56	0	0	0	1	1	16	1	0	0	1	75
318	56	1	0	0	24.80	1	0	0	1	1	16	1	1	1	1	120
319	57	0	1	1	24.28	1	0	0	0	1	42	1	1	1	0	105
320	57	0	0	0	24.00	0	0	0	1	1	24	1	0	0	0	89
321	57	1	0	0	18.69	0	0	0	1	0	18	1	0	0	0	90
322	57	0	0	0	24.03	0	0	0	1	1	16	1	0	0	0	91
323	57	1	0	0	19.83	0	0	0	1	1	29	1	1	0	1	120
324	57	1	0	0	24.44	1	0	0	1	0	12	1	0	1	1	65
325	57	1	0	0	19.56	1	0	0	0	0	20	1	0	1	1	25
326	57	1	1	1	28.03	0	0	0	1	1	28	1	1	0	1	86
327	57	0	0	0	22.31	1	0	0	1	1	19	1	0	0	0	65
328	57	1	0	0	25.28	1	0	0	1	1	20	1	0	0	1	60
329	58	0	0	0	25.60	0	0	0	1	1	25	1	0	0	0	50
330	58	0	1	0	21.91	0	0	0	1	1	15	1	1	0	0	120
331	58	1	0	0	23.46	1	0	0	1	0	18	1	0	1	0	82
332	58	0	0	0	25.06	1	0	0	1	0	26	1	1	1	0	60
333	58	0	0	0	31.11	1	1	0	1	0	18	1	0	0	1	82
334	58	0	0	0	22.38	1	0	0	1	0	18	1	1	1	0	90
335	58	1	1	1	28.41	0	0	0	1	1	19	1	1	1	1	115
336	58	1	0	0	23.31	1	0	0	0	1	27	1	0	0	0	90
337	58	1	0	0	21.87	0	0	0	1	1	25	1	0	0	0	45
338	58	0	0	0	23.83	0	0	0	1	0	17	1	0	0	1	45
339	58	0	0	0	29.75	0	0	0	0	1	15	1	0	0	1	73
340	58	1	0	0	25.93	0	0	0	1	1	17	1	1	0	0	50
341	59	0	0	1	22.41	1	0	0	0	0	17.5	1	0	1	1	50
342	59	0	0	1	22.83	1	0	0	1	0	19	1	1	0	1	121
343	59	0	0	0	21.23	1	0	0	1	1	26	1	1	1	1	50
344	59	1	0	0	23.88	1	0	0	1	0	21	1	0	0	1	35
345	59	1	0	1	28.37	1	0	0	1	0	18	1	0	0	1	59
346	59	1	0	0	27.76	0	0	0	0	1	26	1	1	0	1	171
347	60	1	0	0	19.63	1	0	0	1	1	29	1	1	0	0	95
348	60	0	0	0	25.10	1	1	0	0	0	21	1	0	1	0	70
349	60	1	1	0	26.51	0	1	0	1	1	27	1	0	0	1	110
350	60	1	1	0	22.09	1	0	1	1	0	10	1	1	1	1	55
351	60	1	0	0	20.81	0	0	0	1	0	8	1	1	0	0	45
352	60	1	0	0	18.94	0	0	0	1	1	17	1	1	0	1	25
353	60	1	0	0	25.62	0	0	0	1	1	18	1	1	0	1	60
354	60	0	0	0	24.93	0	0	0	1	0	20	1	0	0	1	120
355	60	0	1	0	24.97	1	0	0	0	1	25	0	0	1	1	60
356	60	1	1	0	26.73	0	0	0	1	1	22	1	1	0	0	73
357	60	1	0	0	22.60	1	0	0	1	1	23	1	0	0	0	90
358	60	1	0	1	23.36	0	0	0	1	0	17	0	0	0	0	90
359	61	1	0	1	20.38	1	0	0	1	1	21	1	0	1	0	105
360	61	0	0	0	19.15	0	0	0	0	0	15	1	1	0	0	65
361	61	1	0	0	21.91	1	0	0	1	1	44	1	0	0	1	105
362	61	0	0	1	19.11	0	0	0	1	0	23	1	0	0	0	105
363	61	0	0	0	23.23	1	0	0	0	0	21	1	1	0	1	40

364	61	1	0	0	27.68	1	0	0	1	0	10	1	0	1	1	120
365	61	1	0	1	22.89	1	0	0	0	1	24	1	1	0	0	60
366	61	0	0	1	22.22	0	0	0	1	0	17	1	1	0	1	60
367	61	1	0	0	19.03	1	0	0	1	0	10	1	0	1	0	65
368	62	0	1	0	22.77	1	1	0	1	0	17	1	0	0	0	50
369	62	1	0	0	23.18	1	0	0	1	0	16	1	0	1	0	50
370	62	0	0	1	22.77	1	0	0	1	0	19	1	0	0	0	110
371	62	0	0	1	28.34	0	0	0	1	0	18	1	1	0	1	66
372	62	0	0	1	27.06	1	1	0	1	1	22	1	1	0	0	75
373	62	1	0	1	24.49	1	0	0	1	1	41	1	1	0	1	65
374	62	0	1	0	19.53	1	0	0	1	1	18	1	0	0	0	80
375	62	1	0	0	25.48	1	0	0	0	0	40	1	0	0	1	100
376	62	0	0	0	24.67	1	1	1	0	0	17	1	1	0	1	77
377	62	0	0	0	20.81	1	0	0	0	0	19	1	0	1	0	90
378	62	0	0	0	31.25	0	0	0	0	0	15	1	0	0	1	103
379	62	0	0	0	22.11	1	0	0	0	0	20	1	1	0	1	125
380	62	0	0	0	22.77	0	0	0	1	1	19	1	0	0	1	120
381	62	0	1	0	27.76	0	0	0	1	0	18	1	0	0	1	54
382	62	0	0	1	24.61	0	0	0	1	0	24	1	0	0	0	130
383	62	0	1	0	24.89	1	0	0	0	1	20	1	1	0	0	78
384	63	1	1	0	20.31	1	0	0	0	1	19	1	1	0	0	60
385	63	0	0	0	25.04	1	0	0	0	0	17	1	0	0	1	55
386	63	0	0	0	24.97	0	0	0	1	0	22	1	0	0	1	60
387	63	1	0	0	21.29	0	0	0	0	0	23	1	0	0	0	50
388	63	0	0	0	21.64	1	1	0	0	0	20	1	0	0	1	106
389	63	1	0	0	26.57	1	0	0	1	0	22	1	0	1	0	70
390	63	0	0	0	23.14	1	0	0	1	0	19	1	1	1	1	120
391	63	1	0	0	26.57	0	0	0	1	0	20	1	1	0	1	70
392	63	1	0	1	23.81	0	0	0	1	1	26	1	0	0	1	48
393	63	1	0	1	24.34	0	0	0	0	0	15	1	0	0	1	69
394	64	0	0	0	15.94	1	0	0	1	0	11	1	1	0	1	115
395	64	0	0	1	25.80	0	0	0	1	0	18	1	0	0	0	62
396	64	1	0	0	21.51	1	0	0	0	0	18	1	0	1	0	80
397	64	1	0	1	23.88	0	0	0	1	0	16	1	0	0	1	70
398	64	1	0	0	16.96	1	0	1	1	1	20	1	0	1	0	120
399	64	1	1	1	26.95	0	0	0	0	1	25	1	1	0	0	115
400	64	1	0	1	26.12	1	0	0	0	0	18	1	0	0	0	65
401	65	1	0	0	25.40	0	0	0	1	0	17	1	0	1	0	40
402	65	1	0	0	23.74	1	0	0	1	0	18	1	1	0	1	25
403	65	0	0	1	24.91	0	0	0	1	0	13	1	0	0	0	40
404	65	0	0	0	19.20	1	0	0	1	0	18	0	0	0	0	75
405	65	1	1	0	25.25	0	0	0	1	1	12	1	1	0	0	40
406	65	1	0	0	23.88	0	0	0	1	1	18	1	1	1	1	75
407	65	1	0	1	23.63	1	0	0	0	0	17	1	1	0	0	80
408	65	1	0	1	22.48	1	0	0	0	0	20	1	0	1	0	80
409	66	0	0	0	17.65	1	0	0	0	0	12	1	1	0	0	90
410	66	0	1	1	33.46	0	0	0	1	0	20	1	0	0	1	55
411	67	0	1	0	27.94	0	0	0	1	1	23	1	1	0	1	114
412	67	0	0	1	28.24	1	1	0	1	0	16	1	0	1	0	43
413	67	1	0	0	23.03	0	0	0	1	1	16	1	1	0	0	75
414	67	1	0	0	26.56	1	0	0	0	1	18	1	1	0	1	76
415	67	1	0	0	24.51	1	0	0	1	0	18	1	0	0	1	57
416	67	0	0	0	26.71	1	0	0	1	1	19	1	1	1	0	60
417	67	1	0	0	22.04	1	1	0	0	0	12	1	1	1	0	40

418	68	0	1	1	25.69	1	1	1	0	1	23	1	0	1	1	40
419	68	1	0	0	16.96	1	0	0	0	1	20	1	0	0	0	120
420	68	1	0	0	17.18	0	0	0	1	0	15	1	0	0	1	75
421	68	1	0	1	26.08	1	0	0	1	1	27	1	1	0	1	65
422	69	1	0	0	19.53	1	0	0	1	1	20	0	1	0	1	88
423	69	1	1	1	15.94	1	0	0	1	0	11	1	1	1	1	115
424	69	1	0	0	23.45	0	0	0	0	0	21	1	0	0	0	95
425	70	0	0	0	22.48	1	0	0	1	0	20	1	1	0	0	80
426	70	1	1	0	24.02	0	0	0	1	1	10	1	0	0	0	128
427	70	1	0	1	26.23	0	0	0	0	1	26	1	0	0	0	71
428	70	1	0	0	28.01	1	0	0	0	0	19	1	1	0	1	100
429	70	1	0	1	22.68	1	1	1	1	1	23	1	0	1	0	67
430	70	1	0	1	22.60	0	0	0	1	0	20	1	0	0	1	75
431	70	1	1	0	27.04	0	0	0	1	1	15	1	1	0	0	60
432	71	1	0	0	24.91	1	0	0	0	0	13	1	1	1	0	40
433	71	1	0	0	21.26	1	0	0	0	0	17	1	1	0	0	90
434	72	1	1	0	2.29	0	0	0	1	1	22	0	1	0	1	70
435	72	1	0	0	25.40	1	0	0	1	0	17	1	0	0	0	40
436	72	1	1	1	29.75	0	0	0	1	1	26	1	0	0	0	78
437	73	1	0	1	23.74	0	0	0	0	0	18	1	1	0	1	25
438	73	0	1	1	23.63	1	0	0	1	0	17	1	0	0	0	80
439	73	1	1	0	21.97	0	0	0	1	1	20	1	1	0	1	62
440	74	1	0	0	17.65	1	0	0	1	0	12	1	1	0	0	90
441	75	1	0	0	25.69	1	1	1	0	0	12	1	0	1	0	40
442	75	1	0	0	22.04	0	0	0	0	1	23	1	1	0	1	40
443	76	0	0	0	25.30	1	0	0	0	1	27	1	0	0	1	130
444	85	0	0	1	16.02	0	0	0	1	1	25	1	1	0	1	105

flexible ureteroscopy for renal stones				
Surgery(0-application of suctioning UAS;1-non-aplication of suctining [illegible]	Residual_stones on 1month postoperatively	Residual_stones-on 1 day postoperatively	SIRS (0-No;1-yes)	Fever(0-No;1-yes)
1	1	1	0	0
1	1	1	0	0
1	1	1	0	0
0	0	0	0	0
1	0	1	0	0
0	0	0	0	0
0	0	0	0	0
1	0	0	0	0
1	1	1	0	0
1	1	1	0	0
1	1	1	0	0
1	1	1	0	0
1	0	0	0	0
1	0	1	0	0
1	1	1	0	0
1	0	0	0	0
1	0	1	0	0
1	1	1	0	0
1	1	1	0	0
1	0	1	0	0
1	0	0	0	0
1	1	1	0	0
1	1	1	0	0
1	1	1	0	0
1	0	0	0	0
0	0	0	0	0
1	1	1	0	0
1	1	1	0	0
1	1	1	0	0
0	0	0	0	0
1	0	0	0	0
1	0	0	0	0
1	0	0	0	0
1	0	1	0	0
1	0	0	0	0
1	0	0	0	0
1	0	0	1	1
1	0	0	0	0
1	0	0	0	0

1	0	0	0	0
1	0	0	0	0
1	0	1	0	0
1	0	0	0	0
1	0	0	0	0
1	0	0	0	0
1	1	1	0	0
1	1	1	0	0
1	0	0	1	1
1	0	0	0	0
0	1	1	0	0
0	0	0	0	0
1	0	0	0	0
1	0	0	0	0
1	0	0	1	0
1	0	0	0	0
1	0	0	0	0
1	0	0	0	0
1	0	0	0	0
1	0	0	0	0
0	0	0	0	0
1	0	0	1	1
1	0	0	0	0
1	0	0	0	0
1	0	0	0	0
1	0	0	0	0
1	0	0	0	0
1	0	0	0	0
1	0	0	1	1
1	1	1	0	1
1	0	0	0	0
0	0	0	0	0
0	0	0	0	0
0	0	0	0	0
0	0	0	0	0
1	0	0	0	0
1	0	0	0	0
1	0	0	0	0
1	0	0	0	0
1	0	0	0	0
1	0	0	0	0
1	1	1	0	0
1	0	0	0	0
1	0	0	0	0
1	1	1	1	0
1	1	1	0	0
1	0	0	0	1
1	0	0	0	0
1	0	0	0	0
1	0	0	0	0
1	0	0	0	0
1	0	0	0	0
0	0	0	0	0
1	0	0	0	0

1	0	0	0	0
1	0	0	0	0
1	0	0	0	0
0	0	0	0	0
0	0	0	0	0
0	0	0	0	0
1	0	0	0	0
1	0	0	0	0
1	1	1	1	0
1	0	0	0	0
1	0	0	0	0
0	0	0	0	0
0	0	0	0	0
1	1	1	1	1
1	0	1	0	0
1	0	0	0	0
1	1	1	0	0
1	0	0	0	0
0	0	0	0	0
0	0	0	0	0
1	0	0	0	0
1	0	0	0	0
1	0	0	0	0
1	0	0	0	0
1	0	0	0	0
0	0	0	0	0
1	0	0	0	0
1	0	0	0	0
1	0	0	1	0
1	0	0	1	1
1	0	0	0	0
1	0	0	0	0
1	0	0	0	0
1	0	0	0	0
1	1	1	0	0
0	0	1	0	0
1	0	0	1	0
1	1	1	1	1
1	0	0	0	0
1	0	0	0	0
1	1	1	0	0
1	0	0	0	0
1	0	0	0	0
1	0	0	0	0
1	1	1	0	0
1	1	1	0	0
1	1	1	0	0
0	0	0	0	0
0	0	0	0	0
1	0	0	0	1
1	1	1	0	0
1	1	1	0	0
1	1	1	1	1
1	0	0	0	0

1	0	0	0	0
1	0	0	1	1
1	0	0	0	0
0	0	0	0	0
0	0	0	0	0
1	0	0	0	0
1	0	0	0	0
1	0	0	0	0
1	0	0	0	0
1	0	0	0	0
1	0	0	0	0
0	0	0	0	0
1	0	0	0	0
1	0	0	0	0
1	1	1	0	0
1	0	0	0	0
1	0	0	0	0
1	0	0	0	1
1	0	0	0	0
1	0	0	0	0
0	1	1	0	0
1	0	0	0	0
1	0	0	0	0
1	0	0	0	0
1	0	0	0	0
1	0	0	1	1
1	0	0	0	0
1	0	0	0	0
1	0	0	1	0
1	0	0	0	0
1	0	0	0	0
1	0	0	0	0
1	1	1	0	0
1	0	0	0	0
1	0	0	0	0
1	1	1	0	0
1	0	0	0	0
1	0	0	1	1
1	0	0	0	0
0	1	1	1	1
0	0	0	0	0
1	0	0	0	0
1	0	0	0	0
1	0	0	0	0
1	0	0	0	0
1	0	0	0	0
1	0	0	0	0
1	1	1	1	1
1	0	0	0	0
1	0	0	0	0
1	0	0	0	0
1	0	0	0	0
1	0	0	0	0
1	0	0	0	0

1	0	0	0	0
1	0	0	0	0
1	0	0	0	0
1	1	1	0	0
0	0	0	0	0
0	0	0	0	1
0	0	0	0	0
0	0	0	0	0
0	0	0	0	0
1	0	0	1	1
1	0	0	0	0
1	0	0	0	0
1	0	0	0	1
1	0	0	0	0
1	0	0	1	0
1	0	0	0	0
1	0	0	0	0
1	1	1	0	0
1	0	0	0	0
1	0	0	0	0
1	0	0	0	1
1	0	0	1	0
0	0	0	0	0
0	0	0	0	0
0	0	0	0	0
0	0	0	0	0
1	0	0	0	0
1	0	0	0	0
1	0	0	0	0
1	0	0	0	0
1	0	0	0	0
1	0	0	0	0
1	0	0	0	0
1	1	1	0	1
1	0	0	0	0
1	0	0	1	1
0	0	0	0	0
0	0	0	0	0
0	0	0	0	0
0	1	1	0	0
0	0	0	0	0
0	0	0	0	0
0	1	1	0	0
1	0	0	0	0
1	0	0	0	0
1	0	0	0	0
1	0	0	1	1
1	0	0	0	0
1	0	0	0	0
1	0	0	1	1
1	0	0	0	0
1	0	0	0	0
1	0	0	0	0
1	0	0	0	0

1	0	0	0	0
1	0	0	0	0
1	0	0	0	0
1	0	0	0	1
1	0	0	0	0
0	0	0	0	0
0	0	0	0	0
0	0	0	0	0
0	0	0	0	0
0	0	0	0	0
1	0	0	0	0
1	0	0	0	0
1	0	0	0	0
1	0	0	0	0
1	0	0	0	0
1	0	0	1	0
1	0	0	0	0
1	0	0	0	0
1	0	1	0	0
1	0	0	0	0
1	0	0	0	0
1	1	1	0	0
1	0	0	0	0
1	0	0	0	0
1	1	1	1	0
1	0	0	0	0
0	0	0	0	0
0	1	1	0	0
1	0	0	0	1
1	1	1	0	0
1	0	0	0	0
1	0	0	0	0
1	1	1	0	1
0	0	0	0	0
0	0	1	0	0
0	0	0	0	0
0	0	0	0	0
1	0	0	0	0
1	0	0	0	0
1	0	0	0	0
1	0	0	0	0
1	1	1	1	0
1	0	0	1	1
1	1	1	0	0
1	0	0	0	0
1	0	1	0	0
1	0	0	0	0
1	1	1	0	0
1	0	0	1	1
1	0	1	0	0
0	1	1	0	1
1	0	0	0	0
1	1	1	0	0
1	0	1	0	0

1	0	0	0	0
1	0	0	0	0
1	0	0	0	0
0	0	0	0	0
0	0	0	0	0
1	0	0	0	0
1	0	0	0	0
1	0	1	1	0
1	0	0	0	0
1	0	0	0	1
1	0	0	0	0
1	0	0	0	0
1	0	0	0	0
1	1	1	0	0
1	0	0	0	0
1	0	0	0	0
0	0	0	0	0
0	0	0	0	0
1	0	0	0	0
1	1	1	0	0
1	0	0	0	0
1	0	0	0	0
1	0	0	0	0
1	0	0	0	0
1	0	1	0	0
1	0	0	0	0
1	0	0	0	0
1	0	0	0	0
0	0	0	0	0
0	0	0	0	0
1	0	0	0	0
1	0	0	0	0
1	1	1	0	1
1	0	0	0	0
1	1	1	0	0
0	0	0	0	0
1	1	1	0	1
1	0	0	0	0
1	0	0	0	0
1	0	0	0	0
1	0	0	0	0
1	0	0	0	0
0	0	0	0	0
0	0	0	0	0
0	0	0	0	0
0	0	0	0	0
0	1	1	0	0
0	1	1	0	0
1	0	0	0	0
1	0	0	0	0
1	0	0	0	0
1	0	0	1	1
1	0	0	0	0
1	0	0	0	0

1	1	1	0	0
1	0	1	0	0
1	0	0	0	0
1	0	0	0	0
1	0	0	1	0
1	0	0	0	0
1	0	0	1	1
1	1	1	0	0
1	1	1	0	0
1	1	1	0	0
1	0	0	0	0
1	0	0	0	1
1	0	0	0	0
1	0	0	0	0
1	0	0	1	0
1	0	0	0	0
0	0	0	0	0
0	0	0	0	0
0	0	0	0	0
1	0	1	0	0
1	0	0	0	0
1	0	0	0	0
1	0	0	0	0
1	0	0	0	0
1	1	1	0	0
1	0	0	0	0
1	1	1	0	1
1	0	0	0	0
0	0	0	0	0
1	0	0	0	0
1	0	0	0	0
1	0	0	0	0
1	1	1	1	0
1	0	0	0	0
1	0	0	0	1
1	0	0	0	0
0	0	0	0	0
1	0	0	0	0
1	0	1	0	0
1	0	0	0	0
1	0	0	0	0
1	1	1	0	0
1	0	0	0	0
1	0	0	0	0
1	0	0	0	1
1	0	0	0	0
1	1	1	0	0
1	0	0	0	0
1	0	0	1	0
1	0	0	0	0
1	0	0	0	0
1	0	0	0	0
1	1	1	0	0
1	0	0	0	1

1	0	0	0	0
1	0	0	0	1
0	0	0	0	0
1	0	0	0	0
1	0	1	0	0
1	0	0	0	0
1	0	0	0	0
1	0	0	0	0
1	1	1	0	0
1	1	1	0	0
1	1	1	0	0
1	1	1	1	1
0	0	0	0	0
0	0	0	0	0
1	1	1	0	0
1	1	1	0	0
1	0	0	0	0
1	0	0	0	0
0	0	0	0	0
1	0	0	0	0
1	0	0	0	0
0	0	0	0	0
1	0	0	0	0
1	0	0	0	0
1	0	0	0	0
1	0	0	0	1
1	0	1	1	1
